# Supplementary material for: uafR: An R package that automates mass spectrometry data processing
Source: PLoS One. 2024 Jul 5;19(7):e0306202. doi: 10.1371/journal.pone.0306202 (PMC11226021; doi:10.1371/journal.pone.0306202)
Supplement: Attachment — Submitted filename: Review PONE-D-24-10543.docx [file pone.0306202.s001.docx]

Dear Editor,

I would like to thank you for the opportunity to review the manuscript sent by you. I would like to start by pointing out that the manuscript describes a potentially very important tool for GC-MS analysis, which could simply the work of analysts around the world. The research is very well described by authors, and altough I was no able to test their software package, I think it is a great addition for researchers everywhere, and the information about it should be available, even moreso as it is available as freeware. As such, I think this manuscript can be published in your journal, but I would suggest a few points to be discussed in order to improve, in my opinion, the reader’s experience with both the manuscript as well as the software package:

1)	While I am not able to discuss any of the programming choices made by the authors, as firstly they are not described (and not relevant) in this manuscript, the description of the functions of the package seem to be very useful to the scientific community. I think what should be described better in the manuscript are the limitations which can occur when using the package, in order to assure that users don’t mistakenly overrely on using it and ignore doublechecking the results. For example, but the authors probably noticed all these things during development and testing of the package, the discussions section should include some recommended thresholds for compound identification for which the user needs to be aware of the risk of misidentification or at least the threshould for which the accuracy should be considered insufficient. It is hard to describe, but I am sure the authors understand that discussing the limitations and issues where attention by the users is needed to avoid errors in result interpretation by the average (overrelying) user is needed, and important.

2)	While the authors tested the package correctly and in a proper manner with the equipment they had available, I think similarly to the previous point, it would be important to shortly discuss (possible) limitations as result of the small number of mass spectrometers for which the package was tested (if I understand correctly all testing was made using Agilent equipment). For example, a discussion (including a warning of sorts) on the fact that the package might have a different accuracy or performance with data from other vendors, newer/older equipment with different resolution, depending on the state of maintenance of the equipment, the importance of calibrating the mass spectrometer properly etc. Of course, these could be considered common sense issues, but given that often the users who overrely most on scientific manuscripts (and take it word-by-word) are user with less experience and/or know-how, I think a few discussion points on what to pay attention to in order to obtain the most reliable results when using this package, would be a big help and could avoid reporting of erroneous results in the (for the most part) important field in which GC-MS is generally used.

In conclusion, I think the manuscript should definetly be published, and I want to congratulate the researchers for their work. Adding a bit more information and “tips&tricks” to the discussions section would however greatly improve the usability and utility of the manuscript, in my opinion.

Best regards,
