# Supplementary material for: uafR: An R package that automates mass spectrometry data processing
Source: PLoS One. 2024 Jul 5;19(7):e0306202. doi: 10.1371/journal.pone.0306202 (PMC11226021; doi:10.1371/journal.pone.0306202)
Supplement: Attachment — Submitted filename: Rebuttal Letter_uafR PLoS One 2024.docx [file pone.0306202.s002.docx]

**Title**

uafR: An R package that automates mass spectrometry data processing

**Authors**

Chase A Stratton^1,4^, Yvonne Thompson^1^, Konilo Zio^2^, William R. Morrison III^3^, and Ebony G. Murrell^1*^

^1^ The Land Institute, 2440 E Water Well Rd, Salina, KS, 67401
^2^ Wooclap SA, 4 rue des Pères Blancs, 1040 Etterbeek, Belgium

^3^ USDA-ARS, Agricultural Research Service, Center for Grain and Animal Health Research, 1515 College Ave, Manhattan, KS 66502 USA

^4^ Delaware State University, 20 Cecil Street, Department of Biology, Dover, DE, 19901

*Corresponding Author. Email: [murrell@landinstitute.org](mailto:murrell@landinstitute.org)

**Response to Reviewers**

**Reviewer comments are quoted in “**plain text” **while our replies are** *italicized***.**

**Editor’s Comments:**

“1. Please ensure that your manuscript meets PLOS ONE's style requirements, including those for file naming. The PLOS ONE style templates can be found at

https://journals.plos.org/plosone/s/file?id=wjVg/PLOSOne_formatting_sample_main_body.pdf and

<https://journals.plos.org/plosone/s/file?id=ba62/PLOSOne_formatting_sample_title_authors_affiliations.pdf>”

“2. Please note that PLOS ONE has specific guidelines on code sharing for submissions in which author-generated code underpins the findings in the manuscript. In these cases, all author-generated code must be made available without restrictions upon publication of the work. Please review our guidelines at https://journals.plos.org/plosone/s/materials-and-software-sharing#loc-sharing-code and ensure that your code is shared in a way that follows best practice and facilitates reproducibility and reuse.”

*All code and associated datasets are available on GitHub along with their documentation. The repository is linked to this manuscript.*

“4. Thank you for stating the following in the Acknowledgments Section of your manuscript:

[The use of trade names is for the purposes of providing scientific information only and does not constitute endorsement by the United States Department of Agriculture. The USDA is an equal opportunity employer. This project was funded by USDA-NIFA projects: #2021-67034-35135 and #2018-67013-27402; and by generous private donations to The Land Institute. In addition, this work was funded, in part, by a United States Department of Agriculture, National Institute of Food and Agriculture, Crop Protection and Pest Management Grant (#2020-70006-33000) and USDA Agricultural Research Service through Congress-appropriated funds.]

We note that you have provided funding information that is not currently declared in your Funding Statement. However, funding information should not appear in the Acknowledgments section or other areas of your manuscript. We will only publish funding information present in the Funding Statement section of the online submission form.

Please remove any funding-related text from the manuscript and let us know how you would like to update your Funding Statement. Currently, your Funding Statement reads as follows:

[This project was funded by USDA-NIFA projects: #2021-67034-35135 and #2018-67013-27402; and by generous private donations to The Land Institute. In addition, this work was funded, in part, by a United States Department of Agriculture, National Institute of Food and Agriculture, Crop Protection and Pest Management Grant (#2020-70006-33000) and USDA Agricultural Research Service through Congress-appropriated funds.]

The funding statement should read as such:

“This project was funded by USDA-NIFA projects: #2021-67034-35135 and #2018-67013-27402; and by generous private donations to The Land Institute. In addition, this work was funded, in part, by a United States Department of Agriculture, National Institute of Food and Agriculture, Crop Protection and Pest Management Grant (#2020-70006-33000), USDA Agricultural Research Service through Congress-appropriated funds, and a National Institute of Health Research Centers for Minority Serving Institutions Grant (U54MD015959).”

*We have removed mention of funding from the Acknowledgements section.*

**Reviewer 1:**

“They also claim applicability to LC-MS data, however failed to present any data to support nor addressed the fundamental difference between GC-MS and LC-MS data that would make the work flow a challenge.”

*We have updated the manuscript to exclude any language on liquid chromatography and have instead kept it focused on gas chromatography. Though the functions/workflow of uafR should in theory apply to liquid chromatography coupled mass spectrometry, as the mass spectrometer produces output in the same format for these tests as for gas chromatography, we agree that it is inappropriate to list it without direct testing. We have also added language in the conclusion that describes how uafR is theoretically applicable for LC-MS data but testing will be required to confirm this (lines: 306-309).*

“The authors compare their process to “manually selecting, integrating and identifying peaks” that fails to compare to any open source or commercial solution. For a non-targeted experiments, it would be rare for a lab not to use some level of processing software.”

*We totally agree; however, uafR works with the data after it has been processed in this manner. The first paragraph of the introduction (lines: 35 – 54) explains how uafR comes after current processing software.*

“The manuscript would be improved greatly by describing the current GC-MS solution and describing how their code improves on their shortcomings.”

*Our software package does not replace current solutions for initial compound screening and tentative identification. It comes after the processing of samples through software like MassHunter (Agilent) and Compound Discoverer (Thermo). The first paragraph of the introduction describes the current pipeline and we have added additional details (lines: 45 - 46) so the reader can investigate available software/resources.*

“The manuscript was not clear on the process of annotation. Was the EI spectra search against NIST library? Was there accurate mass searching used? Or both? If the latter, how was a parent mass obtained? What was the resolution and mass accuracy of the instrument used to collect the dataset described?”

*We have updated paragraph 3 of the introduction (lines:71-72, 74) to include a more general description of the initial processing of samples through peak detection software. The methodology is also stated in the third paragraph of the section labelled “*Testing the Accuracy of the Package*” (lines: 251-274). We have clarified details in this section regarding the resolution and mass accuracy of our instrument (lines: 256 - 259).*

“The author claim “the most accurate compound areas”, however little support to support this claim. This would be to be compared to other peak detection algorithms and not manual process which the author stated is full of bias.”

*The manual processing of samples we are referring to comes after the use of peak detection software. Aligning peaks across samples remains a difficult and manual process – particularly for complex studies with many samples. uafR automates the best current practices for aligning peaks across samples. The additional details added to the introduction (lines: 71-72, 74, 76) clarify that our package is meant to be employed after commercially available peak detection algorithms. This is the part of the process that does currently employ manual processing, and to our knowledge there is no software package or R package that automates this part of the process. Therefore, uafR should be compared to the manual process currently used, not the peak algorithm programs that are used prior to this identification step. We have removed the words “the most accurate” and revised this section of the manuscript to reiterate the part of the chemical ID processing chain the uafR package is designed to address (line: 332).*

“In addition, any comparison to speed needs comparison to other algorithms instead of manual process.”

*Please refer to our previous response. To our knowledge there is no comparable algorithm used in commercial software for GC-MS chemical identification.*

“The size of the datasets used it the examples were relatively small and do not support any claim to ability to handle large datasets.”

*We have added a statement about the need for further testing on larger datasets (lines: 300 - 301). Though we have successfully run the software on a larger experiment with >17,000 tentatively identified molecules, the data are not yet published so cannot be shared alongside the manuscript.*

“The authors presented a process that was built from what appears to be existing code to perform the tasks numerous software packages can do.”

*While is true that uafR uses three packages – webchem to generate chemical IDs (CIDs) from chemical names, ChemmineR to extract structure data from SDF files for the individual chemicals, and fmcsR to calculate Tanimoto similarities for each chemical compound comparison – the rest of the package and its constituent processes are original and the code was written by the authors. We’ve added language/citations for each of these packages in the manuscript (lines: 146 – 148, 179, and 198 – 199). What our uafR package does is take the structures extracted by ChemmineR and compare it to a user-defined library of known compounds using Tanimoto similarities calculated by the fmcsR package on the fly. uafR’s proprietary algorithm, written by the authors, also uses published information on every tentatively identified GCMS compound to extract component areas for all search chemicals specified by the user.*

*The details regarding the algorithm, as well as the raw code for the package, can be found at* github.com/castratton/uafR. *The uafR package, and its original algorithm, is also currently patent pending (Reference #DSU23-41). Inventions that utilize other inventions are neither redundant nor unoriginal. The wheel, the ignition coil, and the internal combustion engine were all invented prior to 1885, but it was not until that year that the modern automobile was invented that utilized these components in combination.*

*The fact that the package can perform some additional functions that other packages can also perform, such as pinging databases and assimilating data into .csv files, also does not preclude it from being a useful package with some unique functions and combinations of options that make it advantageous to use. For example, both the established and widely used “nlme” and “lme4” R libraries run mixed model analyses, but each package also has some unique functions that make one package more useful than the other for certain datasets or situations.*

“There is no evidence to that this process would be applicable to LC-MS data.”

*This is a valid point; If we wish to reference using this package on LC-MS we should include a test of those data. We have updated the manuscript to generally exclude the mention of LC-MS, but include a statement in the Conclusion that, considering the mass spec output would be the same, it may very well be applicable to these data (lines: 310 - 312). Since uafR works on the output from the peak detection/identification software, and the output from these software are the same for GC/LC-MS data, the package should in theory work on LC-MS data. However, this still needs to be tested.*

“Consistently use GC-MS, and m/z should be italicized.”

*We have corrected this throughout the manuscript.*

**Reviewer 2:**

“In the manuscript, the authors did not mention which mass spectrometer was used for the first dataset, only demonstrate the second dataset of GC-MS data from Agilent 5997B mass spectrometer, which is single quadrupole detector. The authors should provide more examples or demonstrations on other mass spectrometers from different vendors or other types of mass spectrometers, like Q-TOF or Orbitrap, even most cutting-edge ones like TimsTOF HT or Astral.”

*The same mass spectrometer was used for both tests. We have updated the “*Testing the Accuracy of the Package*” section for clarity (lines: 256 – 259, 266 – 267) and added a statement about how more tests of additional instruments are warranted (lines: 310 - 315). That said, the output from other mass spectrometers should include the same data for each component, so uafR should apply to outputs from other GC-MS equipment.*

“If uafR compared with commercial softwares, like Compound Discoverer from Thermo, what are the advantages of uafR compared to these commercial softwares?”

*As stated previously, uafR works on GC-MS data after peak identification and compound searching/matching has occurred. uafR takes the output from commercial software to pull out common chemicals across every sample. uafR does this by acquiring published knowledge (including m/z histograms) from online, public databases. To our knowledge there is no comparable software to uafR.*

“It will be great if the authors could address whether the uafR could handle MS2 or MS3 data, additionally whether uafR could handle ion mobility + MS2 data.”

*We intend to extend our algorithm’s functionality for these additional data types as spectral data on PubChem is updated to include them. The algorithm depends on what is currently published to their server but only improves with more accurate data. We have added this to the manuscript (lines 313 – 315).*

**Reviewer 3:**

“The authors have developed a new tool for processing large volumes of GC-LC/MS data in a minimum of time. They validated this tool using 2 different data sets. In addition, they allow everyone to test this tool with other data sets. In addition, the methods applied are well described and rigorous.”

*We thank the reviewer for their comments!*

**Reviewer 4:**

“I would like to start by pointing out that the manuscript describes a potentially very important tool for GC-MS analysis, which could simply the work of analysts around the world.”

*Thank you, we agree!*

“The research is very well described by authors, and altough I was no able to test their software package, I think it is a great addition for researchers everywhere, and the information about it should be available, even moreso as it is available as freeware.”

*We appreciate your support.*

“I think what should be described better in the manuscript are the limitations which can occur when using the package, in order to assure that users don’t mistakenly overrely on using it and ignore doublechecking the results. For example, but the authors probably noticed all these things during development and testing of the package, the discussions section should include some recommended thresholds for compound identification for which the user needs to be aware of the risk of misidentification or at least the threshould for which the accuracy should be considered insufficient. It is hard to describe, but I am sure the authors understand that discussing the limitations and issues where attention by the users is needed to avoid errors in result interpretation by the average (overrelying) user is needed, and important.”

*We thank the reviewer for this comment and we concur. We have added a statement to the conclusion that makes it clear that researchers should still check that the output makes chemical/biological sense (lines: 318 - 323).*

“While the authors tested the package correctly and in a proper manner with the equipment they had available, I think similarly to the previous point, it would be important to shortly discuss (possible) limitations as result of the small number of mass spectrometers for which the package was tested (if I understand correctly all testing was made using Agilent equipment).”

*We agree and have added a direct statement about this limitation. (lines: 310 - 315)*

“Of course, these could be considered common sense issues, but given that often the users who overrely most on scientific manuscripts (and take it word-by-word) are user with less experience and/or know-how, I think a few discussion points on what to pay attention to in order to obtain the most reliable results when using this package, would be a big help and could avoid reporting of erroneous results in the (for the most part) important field in which GC-MS is generally used.”

*We added a statement after advising researchers to validate that the output makes chemical/biological sense about how categorate() can be used in the validation (lines: 321 - 323). The output includes chemical, biological, and structural information (among additional details) for every molecule in a study. The alternative would be looking up every molecule one by one on relevant databases.*

“Adding a bit more information and “tips&tricks” to the discussions section would however greatly improve the usability and utility of the manuscript, in my opinion.”

*We thank the reviewer for their feedback and consistency with the suggestions. We believe the manuscript is much improved by adding these components.*

**Reviewer 5:**

“I have previously reviewed this manuscript for a different journal and during the multiple revisions reviewed concluded that the manuscript was ready for publication. Only minor changes have been made since, so I can still recommend this for publication as is.”

*We would like to thank the reviewer for their consistently thorough review of uafR. Their feedback on the previous round of edits was well received and significantly improved the manuscript.*

“The only comment I'll make here is that the authors could tone down a bit of language used for more succinct and detail-oriented descriptions of implementation and results (for example, lines 235-237).”

*We have done our best to tone down the language and focus on the details.*

**Reviewer 6:**

“I can only state that there is a need for such informatic tools but I cannot state if this one is efficient or not.”

*We appreciate your honesty and agree that there is a need for software such as uafR to fill this current gap in chemical research.*
